# Efficacy and toxicity of treatment of smoldering multiple myeloma: a systematic review and meta-analysis

**DOI:** 10.1080/07853890.2025.2560679

**Published:** 2025-09-24

**Authors:** Lixin Quan, Qun Li, Chi Ma, Xinxin Cao, Chunyan Sun

**Affiliations:** ^a^ Institute of Hematology, Union Hospital, Tongji Medical College, Huazhong University of Science and Technology, Wuhan, China; ^b^ Department of Hematology, Peking Union Medical College Hospital, Chinese Academy of Medical Sciences and Peking Union Medical College, Beijing, China

**Keywords:** Smoldering multiple myeloma, efficacy, safety, early treatment, meta-analysis

## Abstract

**Objective:**

Smoldering multiple myeloma (SMM) is a slowly progressive, asymptomatic plasma cell disorder. This meta-analysis evaluated the efficacy and safety of novel agent-based therapies for SMM, particularly high-risk patients.

**Methods:**

PubMed, Embase, Web of Science, Ovid MEDLINE, Scopus and ClinicalTrials.gov were searched for randomized controlled trials (RCTs) and non-randomized studies from 2003 to 2024.

**Results:**

Nineteen studies were included, comprising 5 RCTs and 14 non-randomized, single-arm trials. These studies involved a total of 1217 patients. Pooled analysis of intervention groups showed progression-free survival (PFS) and overall survival (OS) rate at 12 months of 94% (95% CI, 89%–98%) and 99% (95% CI, 97%–100%), respectively. The overall response rate (ORR) was 64% (95% CI, 50%–77%), and the complete response rate (CRR) was 12% (95% CI, 3%–25%). Minimal residual disease (MRD) negativity rate was 62% (95% CI, 42%–81%), and grade 3–4 adverse events (AEs) rate was 36% (95% CI, 30%–43%). As a comparison, we pooled data from the control groups of 5 RCTs, showing that PFS and OS rate-12m were 76% and 97%, respectively, with 0% CRR, 0% ORR and 25% grade 3–4 AEs. Subgroup analysis revealed high-risk SMM patients achieved higher PFS rate −12 m (97% vs. 91%), ORR (77% vs. 53%) and CRR (24% vs. 5%) compared to all SMM patients, with similar OS rate-12m (99% vs. 99%) and AEs rates (38% vs. 34%).

**Conclusion:**

Early intervention may delay progression and improve clinical responses, especially in high-risk SMM. However, adverse events (AEs) warrant caution. More well-designed RCTs are needed to confirm our findings.

## 1. Introduction

Smoldering multiple myeloma (SMM) is a slowly progressive, asymptomatic plasma cell disorder. It is the intermediate disease stage between monoclonal gammopathy of undetermined significance (MGUS) and multiple myeloma (MM). The terminology of ‘smoldering myeloma’ was first proposed by Kyle et al. in 1980 [1] to describe six patients with elevated bone marrow plasma cells (BMPC) and serum monoclonal protein but without end-organ damage. Up to now, the definition of SMM has gone through a series of developments, based on the International Myeloma Working Group (IMWG) diagnostic criteria published in 2014, SMM is defined by serum monoclonal protein (IgG or IgA) ≥30 g/L or urinary monoclonal protein ≥500 mg per 24 h and/or clonal bone marrow plasma cells 10–60% and with the absence of myeloma defining events or amyloidosis [2]. Previous studies have shown that the progression rate of SMM to MM is around 10% annually per year over the first 5 years after diagnosis, decreasing to 3% per year over the next 5 years, and further dropping to 1% per year thereafter [3]. Once the disease progresses to multiple myeloma, it is almost impossible to escape the fate of recurrence, even if treated promptly. In addition, since high-risk SMM (HRSMM) are more likely to experience disease progression and greater disease burden than patients with low- or intermediate-risk SMM, several works of literature suggest that SMM patients with multiple-risk factors might derive benefit from early intervention [4]. However, according to Kakkilaya et al. there was no convincing evidence to suggest that SMM exhibited a greater responsiveness to therapeutic interventions compared to MM [5]. Therefore, investigating whether early intervention can effectively halt disease progression in SMM patients, particularly those with HRSMM, remains crucial.

Even though the risk stratification of SMM has been continuously evolving over the past forty years [6], optimal monitoring is still the prevailing standard care for SMM. Some HRSMM patients may be enrolled in clinical trials to evaluate the early treatment, defined as initiating therapy immediately after diagnosis. Traditional treatments such as melphalan and prednisone have been shown to have no benefit in overall survival for early therapy and have major side effects [7–9]. Furthermore, SMM is a kind of heterogeneous disease and there are two subsets of patients: one is with biological premalignancy, and the other is with CRAB-negative malignancy [2]. For patients similar to MGUS, it is important to avoid overtreatment, emotional stress and financial loss. However, for HRSMM patients with a relatively greater tendency to progress, early intervention may bring significant benefits. At present, more and more novel drugs are being applied to MM and have achieved great results; many studies have begun to test the efficacy and toxicity of these drugs in SMM. Research into early treatment in SMM has identified two main strategies: one is aimed at controlling the disease and delaying the progression time to MM, using a low-intensity intervention, such as lenalidomide; the other with a high-intensity regimen is aimed at curing since the mutation burden of SMM is lower than MM so that the disease may be more sensitive to treatment and cure may be achieved by inducing a deep response [10]. Which kind of strategy is appropriate remains controversial. A meta-analysis included eight randomized clinical trials (RCTs), three of which were on MP, and two were on bisphosphonates; however, only three RCTs contained novel drugs that are effective in treating MM [9]. Many single-arm clinical trials are currently exploring the dosage and effect of various regimens on SMM, and these trials make great contributions to the treatment of SMM. Therefore, including more clinical trials is of great importance to reassess the intervention. Furthermore, drug-resistant patients also need to be taken into account when choosing different treatment regimens. A stepwise escalation design may reduce selective pressure on tumour subclones, thereby postponing or averting the development of acquired resistance [11]. To evaluate the benefit and risk of early intervention versus observation of SMM, and the two intervention strategies, we conducted this meta-analysis including 4 RCTs and 15 non-randomized studies of interventions (NRSI). In performing the meta-analysis, we conducted the subgroup analysis based on study type, patient’s risk stratification, treatment purpose and agent type.

## 2. Material and methods

This meta-analysis complied with the Preferred Reporting Items for Systematic Reviews and Meta-Analyses (PRISMA) and has been registered in Prospero (CRD420250505462).

### 2.1. Search strategy

We performed a comprehensive search in the databases PubMed, Embase, Web of Science, Scopus, ClinicalTrials.gov and Ovid MEDLINE, covering randomized controlled trials and non-randomized studies of interventions published in English between 2003 and 2024.3.13. The start year was chosen because novel agents began to be introduced. The search terms including MeSH words and entry terms for ‘smoldering multiple myeloma’, ‘Clinical trial’, ‘intervention’, ‘therapy’, ‘drug treatment’, ‘Lenalidomide’, ‘Daratumumab’, ‘Ixazomib’, ‘Isatuximab’, ‘Elotuzumab’, ‘Pembrolizumab’, ‘Carfilzomib’, ‘Siltuximab’, ‘Ibrutinib’, ‘Thalidomide’.

### 2.2. Selection criteria

Studies satisfying the following criteria were selected for inclusion in the meta-analysis.

1. Participants: SMM patients were adult patients diagnosed with smoldering multiple myeloma (using any recognized diagnostic criteria). HRSMM patients were adult patients diagnosed with high-risk smoldering multiple myeloma (using PETHEMA 2007 [12], Mayo 2008 [13], IMWG 2010 [14], Rajkumar et al. 2015 [15] or Mayo 2018 criteria [16]).
2. Interventions: Treatment arm included any drug treatment (Lenalidomide, Daratumumab, Ixazomib, Isatuximab, Elotuzumab, Pembrolizumab, Carfilzomib, Siltuximab, Ibrutinib and Thalidomide) whether or not a control group had been implemented.
3. Outcomes: Outcome data comprised progression-free survival (PFS), overall survival (OS), overall response rate (ORR), complete response rate (CRR), minimal residual disease (MRD) negativity rate and any grade 3–4 adverse events (AEs) rate.
4. Study design: Prospective open-label NRSIs and RCTs.

### 2.3. Data extraction

Two reviewers independently extracted the information from the eligible studies using Microsoft Excel: basic information containing the name of the first author, year of publication, study design, treatment regimen, a number of patients and risk stratification of patients. We extracted relevant data from intervention groups and control groups from RCTs or single-arm trials. Indicators evaluated efficacy and safety including PFS rate-12m, OS rate-12m, ORR, CRR, MRD-Negative Rate, and any grade 3–4 AEs rate. The published Kaplan–Meier (K-M) survival curves were used to extract 12-month PFS and OS data points using Origin software. For cohorts with multiple available reports, we prioritized the latest publication for data acquisition. Conflicts were addressed by discussing the issues or consulting other researchers.

### 2.4. Quality assessment

The methodological quality of included RCTs was evaluated with the Cochrane Risk of Bias Tool (RoB 2), whereas NRSIs were assessed using the Methodological Index for Non-Randomized Studies (MINORS) criteria. We evaluated the publication bias by Funnel plot and the Egger test. Conflicts were addressed by discussing the issues or consulting other researchers.

### 2.5. Statistical analysis

Given the inclusion of both RCTs and single-arm trials in our analysis, we pooled the intervention group data from all RCTs and single-arm trials, and then pooled the data of the control groups of all RCTs as a comparison. Subgroup analysis was conducted to determine whether the estimates of effect for the outcomes were influenced by type of study (RCTs vs. single-arm trials), patient’s risk stratification (all SMM vs. HRSMM), treatment purpose (cure vs. control) and treatment regimen (monotherapy vs. combination therapy). We defined the treatment with the aim of cure as an intervention involving three agents with carfilzomib or the use of four agents. The treatment that did not meet the above standards was with the aim of control. Monotherapy was defined as a treatment regimen that included only one therapeutic agent, excluding dexamethasone and bisphosphonates. Combination therapy was defined as a treatment regimen that included two or more therapeutic agents, excluding dexamethasone and bisphosphonates. Within the monotherapy group, treatment regimens were further subdivided into immunomodulatory drugs (IMiDs) (thalidomide or lenalidomide) and monoclonal antibodies (mAbs) (elotuzumab or isatuximab or pembrolizumab or daratumumab). In this meta-analysis, Stata (version 17.0, StataCorp LLC, College Station, TX, USA) was used to analyse relevant data. The statistical heterogeneity will be measured by the Q and I^2^ statistical tests. For the pooled effect size of various ratios (ORR, PFS rate, OS rate, AEs rate), we collected the event rates and the number of participants to evaluate, and the double arcsine transformation was applied to data which was more than 0.8 or less than 0.2. If I^2^>50%, the random-effects model should be applied; otherwise, we will use the fixed-effects model. According to the Egger test, we will evaluate the publication bias. A *P*-value of 0.05 indicates Statistical significance. We performed the sensitivity analysis using the ‘leave-one-out’ method, with significance defined as a change in the pooled effect estimate exceeding 10% when any single study was excluded.

## 3. Results

### 3.1. Study selection

**Figure 1.**
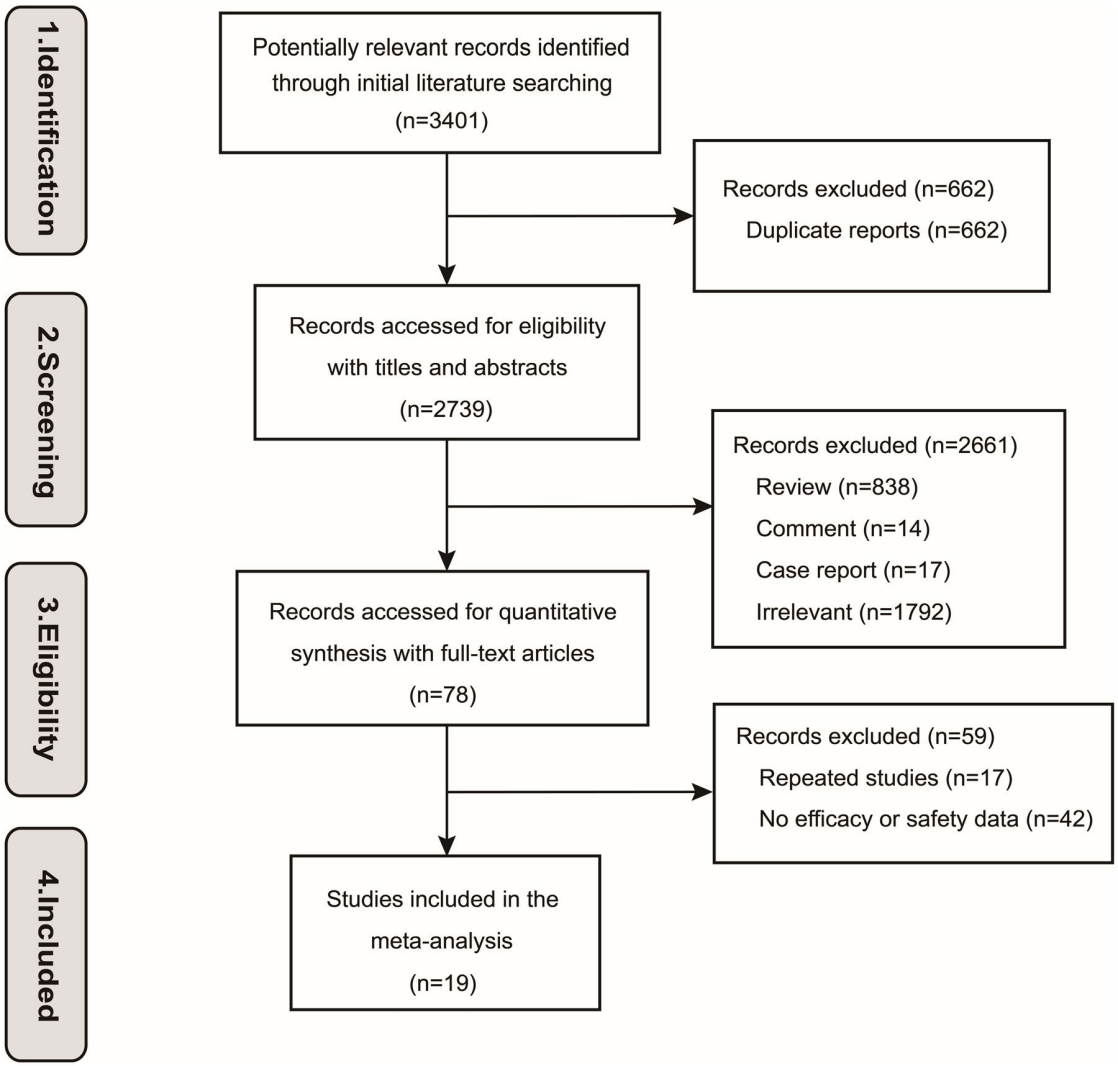
Study flow diagram.

In this comprehensive review, we initially identified a total of 3,401 studies (Figure 1) for inclusion. After a meticulous selection process, which included the exclusion of 662 duplicate studies and 2,661 studies by screening title and abstract, 78 studies were reviewed by full text. On the full-text evaluation of 78 studies, 59 were excluded (17 studies repeatedly reported results from the same clinical trial at different times, and 42 studies included no efficacy or safety data). The remaining 19 studies encompassing 1,217 participants were eligible for our analysis. The studies comprised 5 RCTs and 14 single-arm trials. The study of Lonial et al. 2020 included phase 2 and phase 3 trials, so we divided it into one RCT and one single-arm trial. As for the study by Sklavenitis-Pistofidis et al. 2022, since arm B was halted early (steroid exposure showed little variation between the two arms, due to the premedication requirement for elotuzumab infusion.), we combined arm A and arm B as single-arm trials for analysis instead of RCT. Two non-randomized studies (Jagannath et al. 2018; Landgren et al. 2020) conducted group studies through different doses of the drug, so we broke them down into two or three groups of single-arm trials for analysis. The study by Manasanch et al. 2023 conducted group studies through different interventions; however, the isatuximab group was not included in our study because of insufficient data. The number of trials in our study is calculated based on the split studies (Table 1). The original data of studies included in our study are presented in Table S1.

**Table 1.** Description of articles selected for the meta-analysis.

Study, year	Study type	Sample size (I/C)	Treatment arm /control arm	Treatment purpose	Treatment regimen	Disease	Outcome
Witzig et al. 2013 [17]	RCT	35/33	ThaZol / Zol	control	monotherapy (IMiDs)	SMM	CRR, ORR, PFS, OS, AEs
Mateos et al. 2016 [18]	RCT	57/62	LenDex/Observation	control	monotherapy (IMiDs)	HRSMM^a,b^	CRR, ORR, PFS, OS, AEs
Lonial et al. 2020 [19]	RCT	90/92	Lenalidomide/Observation	control	monotherapy (IMiDs)	SMM	CRR, ORR, PFS, OS, AEs
	SA	44	Lenalidomide	control	monotherapy (IMiDs)	SMM	CRR, ORR, PFS, OS, AEs
Rajkumar et al. 2003 [20]	SA	29	Thalidomide	control	monotherapy (IMiDs)	SMM	CRR, ORR, PFS, OS, AEs
Barlogie et al. 2008 [21]	SA	76	ThaBPs	control	monotherapy (IMiDs)	SMM	CRR, ORR, PFS, OS
Brighton et al. 2019 [22]	RCT	43/42	Siltuximab/Placebo	control	monotherapy (mAbs)	HRSMM^b^	PFS, OS, AEs
Jagannath et al. 2018 [23]	SA	15	Elotuzumab 20 mg/kg	control	monotherapy (mAbs)	HRSMM^c^	CRR, ORR, PFS, AEs
	SA	16	Elotuzumab 10 mg/kg	control	monotherapy (mAbs)	HRSMM^c^	CRR, ORR, PFS, AEs
Manasanch et al. 2019A [24]	SA	24	Isatuximab	control	monotherapy (mAbs)	SMM	CRR, ORR
Manasanch et al. 2019B [25]	SA	13	Pembrolizumab	control	monotherapy (mAbs)	SMM	CRR, ORR, PFS, OS, MRD
Landgren et al. 2020 [26]	SA	41	Daratumumab(Intense)	control	monotherapy (mAbs)	SMM	CRR, ORR, PFS, AEs
	SA	41	Daratumumab(Intermediate)	control	monotherapy (mAbs)	SMM	CRR, ORR, PFS, AEs
	SA	41	Daratumumab(Short)	control	monotherapy (mAbs)	SMM	CRR, ORR, PFS, AEs
Mailankody et al. 2022 [27]	SA	14	IxaDex	control	monotherapy	HRSMM^a,b^	CRR, ORR
Korde et al. 2015 [28]	SA	12	KRd-R	cure	combination	HRSMM^a,b^	CRR, ORR, PFS, OS, MRD
Liu et al. 2018 [29]	SA	49	EloLenDex	control	combination	SMM	CRR, ORR
Mateos et al. 2021 [30]	SA	90	KRd-ASCT-KRd-Rd	cure	combination	HRSMM^a,b^	CRR, ORR, PFS, OS, MRD
Kazandjian et al. 2021 [31]	SA	54	KRd-R	cure	combination	HRSMM^a,b,d^	CRR, ORR, PFS, OS, AEs, MRD
Sklavenitis-Pistofidis et al. 2022 [32]	SA	51	EloLenDex	control	combination	HRSMM^d^	CRR, ORR, PFS, OS
Kumar et al. 2022 [33]	SA	87	DKRd-DKRd-DR	cure	combination	SMM	CRR, ORR, PFS, MRD
Nadeem et al. 2023 [34]	SA	30	DRVd	cure	combination	HRSMM^a,b,d,e^	CRR, ORR, MRD
Manasanch et al. 2023 [35]	SA	36	IsaLen	control	combination	HRSMM^a^	CRR, ORR, AEs

**Note:** Treatment purpose of cure was defined as intervention involving 3 agents with carfilzomib or the use of 4 agents. The treatment that did not meet the above standards was with the aim of control. Monotherapy was defined as a treatment regimen that included only one therapeutic agent, excluding dexamethasone and bisphosphonates. Combination therapy was defined as a treatment regimen that included two or more therapeutic agents, excluding dexamethasone and bisphosphonates. The high-risk stratification was defined according to the following criteria: ^a^ETHEMA 2007 [12]. ^b^Mayo 2008 [13]. ^c^IMWG 2010 [14]. ^d^Rajkumar et al. 2015 [15]. ^e^Mayo 2018 [16].

**Abbreviations:** AEs, adverse events; ASCT, autologous stem cell transplantation; CRR, complete response rate; DKRd, daratumumab–carfilzomib–lenalidomide–dexamethasone; DRVd, daratumumab–lenalidomide–bortezomib–dexamethasone; EloLenDex, elotuzumab–lenalidomide–dexamethasone; HRSMM, high-risk smoldering myeloma; IMiDs, immunomodulatory drugs; IsaLen, isatuximab–lenalidomide; IxaDex, ixazomib–dexamethasone; KRd, carfilzomib–lenalidomide–dexamethasone; LenDex, lenalidomide–dexamethasone; mAbs, monoclonal antibodies; MRD, minimal residual disease; ORR, overall response rate; OS, overall survival; PFS, progression-free survival; SA, single-arm; SMM, smoldering myeloma; ThaBPs, thalidomide–bisphosphonates; ThaZol, thalidomide–zoledronic acid.

### 3.2. Quality of studies

According to RoB 2 tool, 2 RCTs have a low risk of bias, 2 RCTs revealed some concerns and 1 RCT seems to have a high risk of bias (Figure S1). The predominant quality concerns come from the randomization process and deviation from the intended interventions. Some studies do not give the specific process of randomization. As for non-randomized studies, nine studies scored between 12 and 16, which suggests good quality, and the other five studies scored 10 (Table S2).

### 3.3. Efficacy assessment

**Figure 2.**
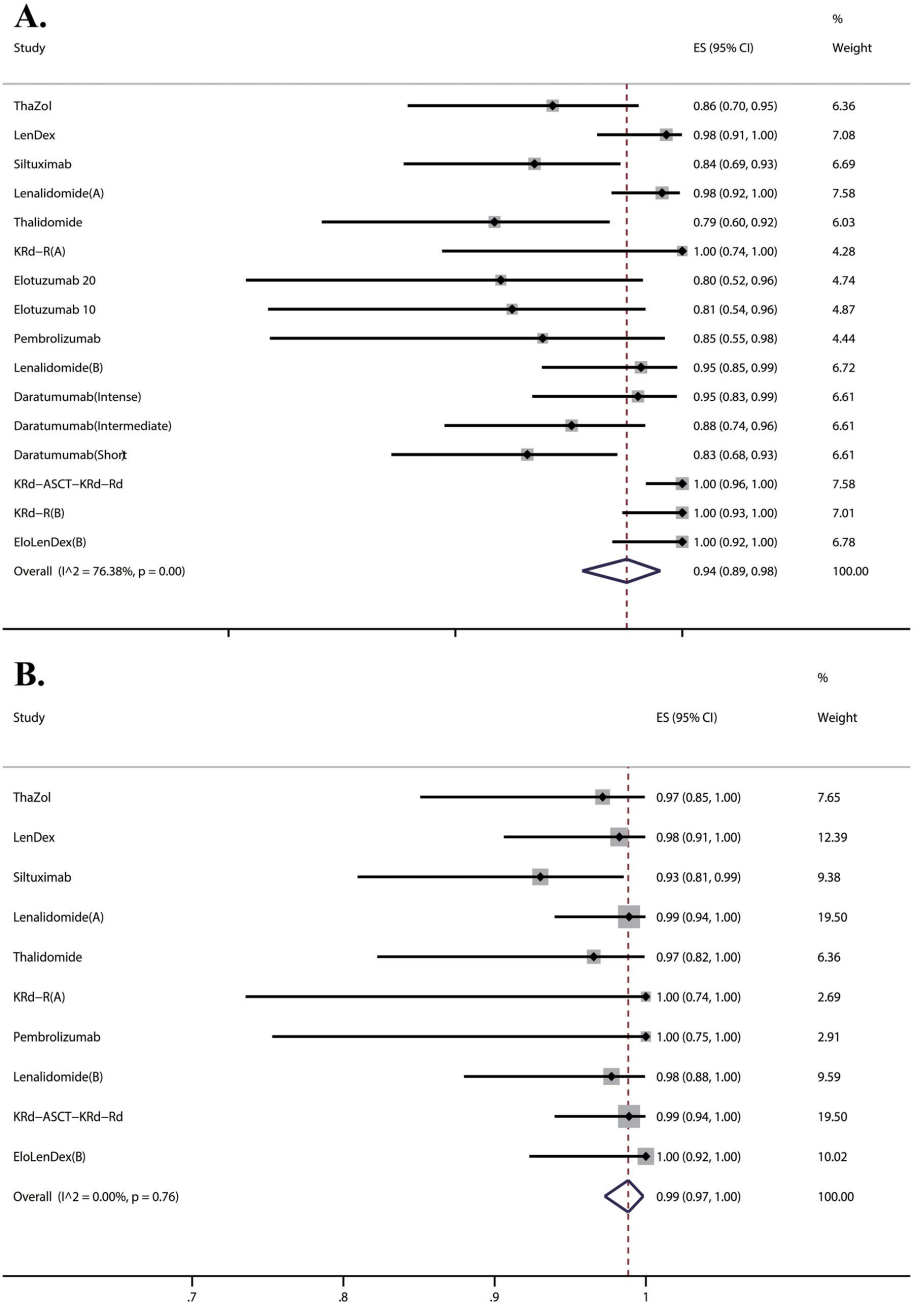
Efficacy assessment of treatment in SMM. (A) PFS rate −12 m of treatment in SMM; (B) OS rate −12 m of treatment in SMM.

To enhance readability, within the meta-analysis figures, we designated the literature by the treatment arm of each study. Lenalidomide (A) and (B) were used to denote RCT and the single-arm study within the Lonial et al.’s 2020 research, respectively. EloLenDex (A) and (B) were utilized to represent the studies conducted by Liu et al. 2018, and Sklavenitis-Pistofidis et al. 2022, respectively. KRd-R(A) and (B) were designated to reference the research articles authored by Korde et al. in 2015 and Kazandjian et al. in 2021. Intervention groups of 4 RCTs and 12 single-arm trials reported the progression-free rate at 12 m (PFS rate –12 m) involving 667 patients. The pooled PFS rate-12m reached 94% (95% CI, 89%–98%, I^2^ = 76%) (Figure 2). Results from the Egger test suggested significant publication bias (*p* < 0.05). I^2^ = 74% means high heterogeneity;therefore, to further explore sources of high heterogeneity, we performed our sub-group analysis by type of study (RCTs or single-arm trials), treatment purpose (cure or control) and treatment regimen. The pooled PFS rate-12m for RCTs was 93% (95% CI, 84%–99%, I^2^ = 77%), compared to 94% (95% CI, 87%–99%, I^2^ = 78%) for single-arm trials. However, the heterogeneity is still high between both groups of studies, so study type was not a factor in heterogeneity. A similar picture emerged in the subgroup analysis of the ‘control’ group and the ‘cure’ group. Patients aiming for cure generally exhibited a higher PFS rate-12m compared to those aimed at controlling disease (Figure S2). According to treatment regimens, 16 studies were categorized into monotherapy and combination therapies, with further subgroup analysis within the monotherapy group. The results indicated that combination therapy had the pooled PFS rate-12m of 100%, higher than the 90% rate with monotherapy (Figure S3). The pooled PFS rate-12m of IMiDs was found to be 93%, while that of mAbs was 87%. In comparison, control groups of 4 RCTs reported a PFS rate-12m involving 229 patients. The pooled PFS rate-12m of the control groups of 4 RCTs was 76% (95% CI, 63%–89%, I^2^ = 81%).

Intervention groups of 4 RCTs and 6 single-arm trials reported the OS rate at 12 m (OS rate-12m) involving 459 patients. The pooled OS rate-12m was 99% (95% CI, 97%–100%, I^2^ = 0%) (Figure 2). Results from the Egger test suggested significant publication bias (*p* < 0.05). Eight studies showed very low heterogeneity; on this basis, we performed our sub-group analysis by study type, treatment purpose and treatment regimen. Results observed in subgroups showed concordance with the overall study findings (Table 2). In comparison, control groups of 4 RCTs reported the OS rate-12m involving 229 patients. The pooled OS rate-12m of the control groups of 4 RCTs was 97% (95% CI, 91%–100%, I^2^ = 72%).

**Table 2.** Subgroup analysis of PFS rate-12m, OS rate-12m, ORR, CRR, any grade 3–4 AEs of treatment in SMM.

Subgroups			PFS rate-12m			OS rate-12m			ORR			CRR			Any grade 3–4 AEs		
			No	ES (95%CI)	P	No	ES (95%CI)	P	No	ES (95%CI)	P	No	ES (95%CI)	P	No	ES (95%CI)	P
Study type	RCT		4	93 (84–99)	<0.01	4	98 (95–100)	0.37	3	56 (33–79)	NA	3	2 (0–14)	NA	4	39 (29–49)	0.08
	SA		12	94 (87–99)	<0.01	6	100 (98–100)	0.85	19	65 (49–80)	<0.01	19	15 (4–30)	<0.01	9	35 (26–44)	<0.01
Treatment purpose	cure		3	100 (99–100)	NA	2	100 (97–100)	NA	5	97 (92–100)	0.05	5	69 (54–83)	<0.01	NA	NA	NA
	control		13	91 (86–96)	<0.01	8	98 (97–100)	0.62	17	51 (39–64)	<0.01	17	2 (1–5)	0.01	NA	NA	NA
Treatment regimen	combination therapy		4	100 (99–100)	0.95	3	100 (98–100)	NA	8	93 (88–97)	<0.01	8	42 (17–69)	<0.01	2	42 (32–52)	0.45
	monotherapy		12	90 (85–95)	<0.01	7	98 (96–100)	0.75	14	43 (32–54)	<0.01	14	2 (0–5)	0.01	11	35 (28–43)	<0.01
		IMiDs	5	93 (86–98)	0.01	5	98 (96–100)	0.88	6	51 (34–67)	<0.01	6	2 (0–7)	<0.01	5	36 (27–46)	0.03
		mAbs	7	87 (82–92)	0.52	2	96 (88–100)	NA	7	34 (18–51)	<0.01	7	3 (0–7)	0.28	6	34 (22–46)	<0.01

**Abbreviations:** AEs, adverse events; CRR, complete response rate; IMiDs, immunomodulatory drugs; mAbs, monoclonal antibodies; ORR, overall response rate; OS, overall survival; PFS, progression-free survival; SA, single-arm.

**Figure 3.**
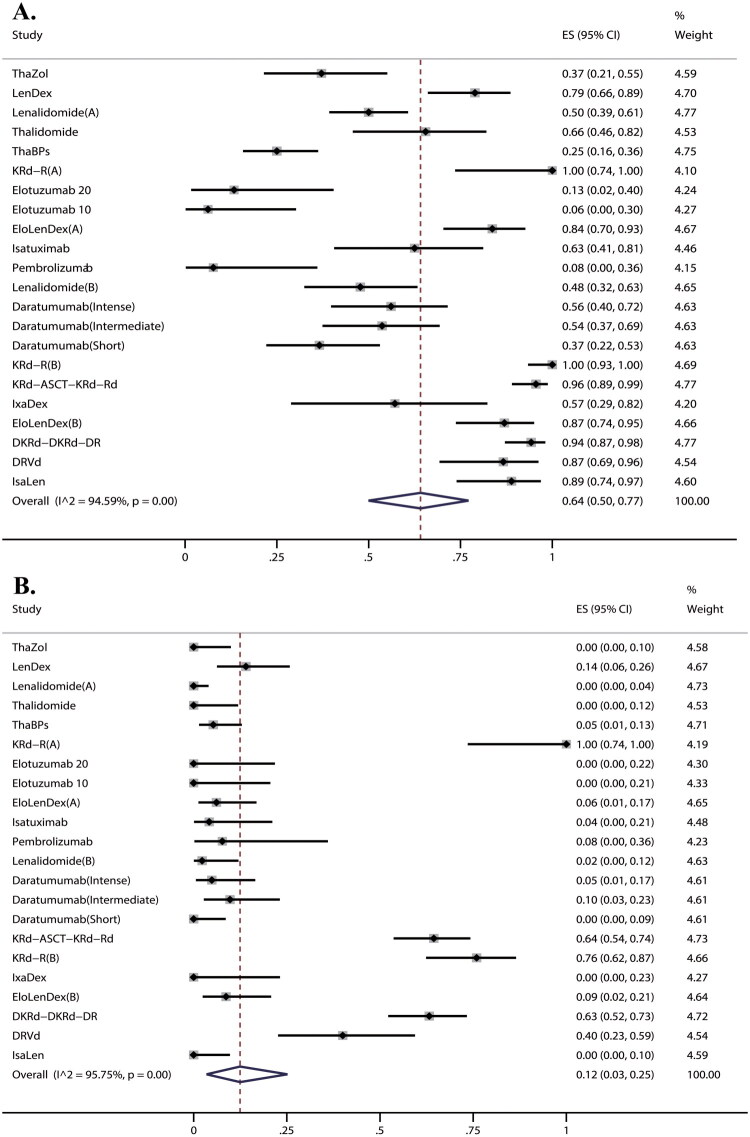
Efficacy assessment of treatment in SMM. (A) ORR of treatment in SMM; (B) CRR of treatment in SMM.

Intervention groups of 3 RCTs and 19 single-arm trials reported the ORR involving 940 patients. The pooled ORR was found to be 64% (95% CI, 50%–77%, I^2^ = 95%) (Figure 3). Results from the Egger test suggested significant publication bias (*p* < 0.05). For ORR we also performed sub-group analysis by type of study, treatment purpose and treatment regimen. Figure S4-6 listed the results of sub-group analysis. We found that patients with the aim of cure showed a better response than those with the aim of controlling disease (97% vs. 51%). Of note, the findings indicated that combination therapy had a significantly higher pooled ORR of 93%, compared to 43% for monotherapy. Within monotherapy, the IMiDs group exhibited a pooled ORR of 51%, while the mAbs group showed a pooled ORR of 34%. In comparison, the control groups of 3 RCTs reported an ORR of 0% involving 187 patients.

**Figure 4.**
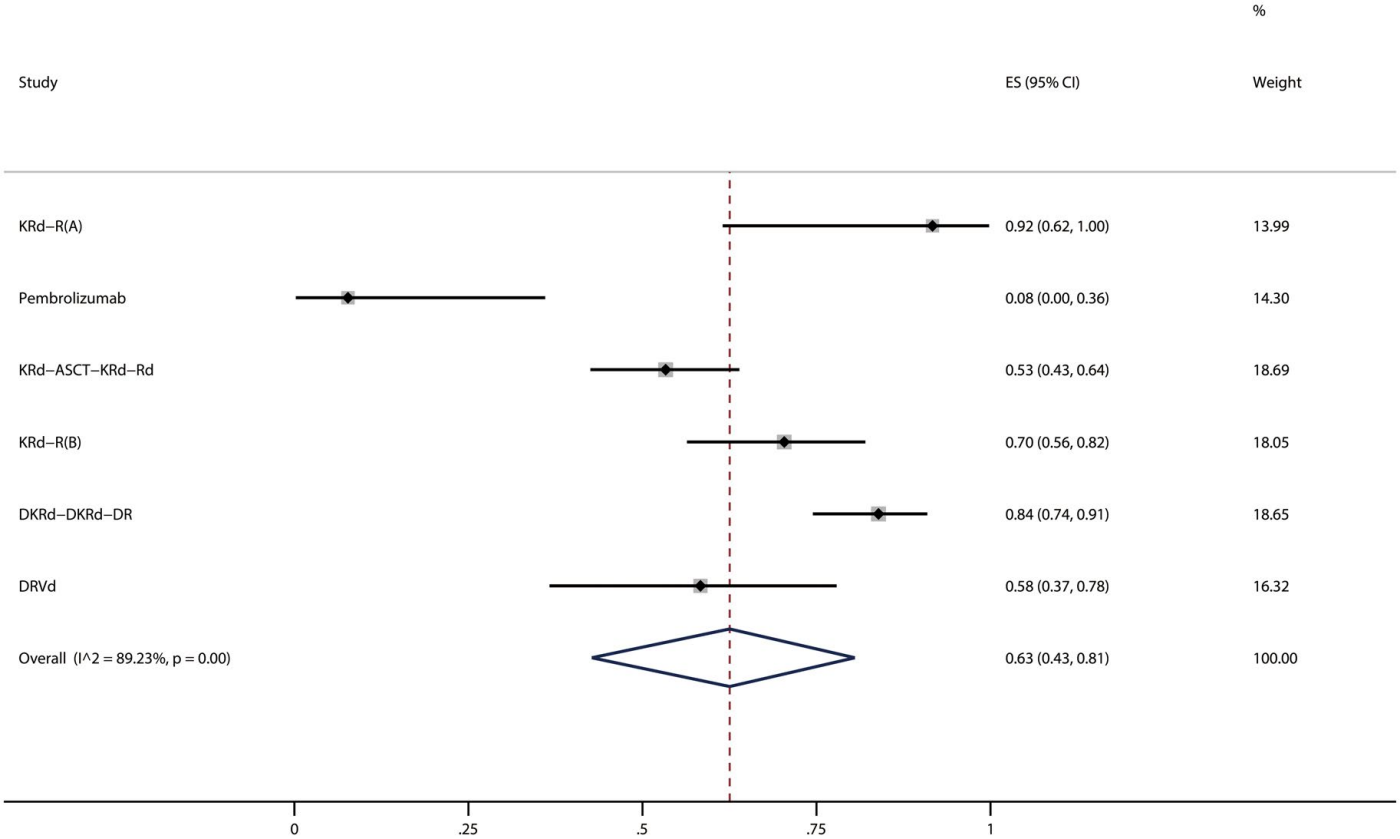
MRD-Negative rate of treatment in SMM.

**Figure 5.**
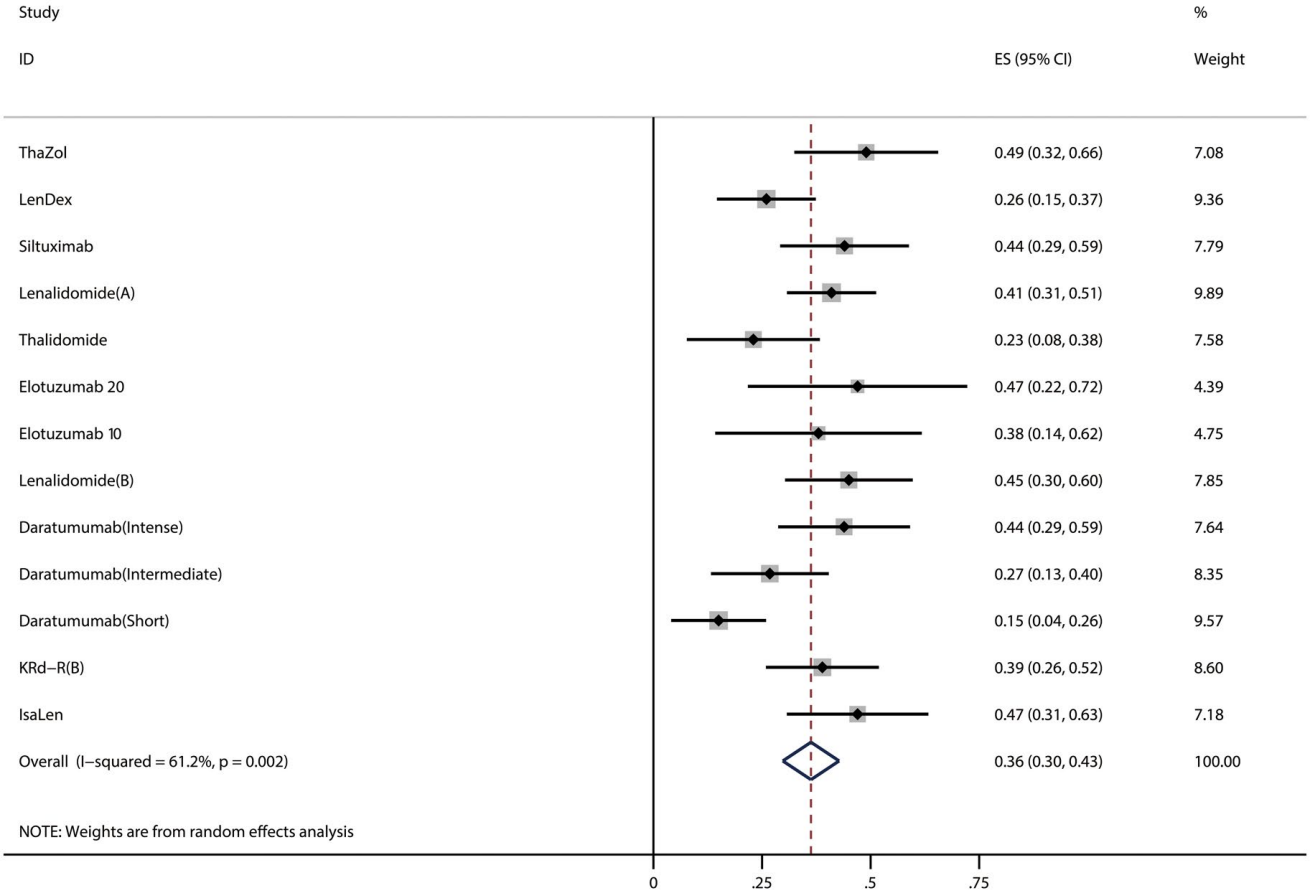
Any grade 3 –4 AEs rate of treatment in SMM.

Intervention groups of 3 RCTs and 19 single-arm trials reported the CRR involving 940 patients. Results indicated a pooled CRR of 12% (95% CI, 3%–25%, I^2^ = 96%) (Figure 3). Results from the Egger test suggested significant publication bias (*p* < 0.05). By subgroup analysis, the pooled CRR of the ‘control’ group was found to be 2% (95% CI, 1%–5%, I^2^ = 52%), and the ‘cure’ group was 69% (95% CI, 53%–81%, I^2^ = 81%), which reflected the notable distinction between the two groups (Figure S7). In addition, combination therapy had a higher pooled CRR of 42%, compared to 2% for monotherapy (Figure S8). The rest of the data is shown in Table 2. In comparison, the control groups of 3 RCTs reported a CRR of 0% involving 187 patients.

All subgroup analysis results are presented in Table 2.

Six single-arm trials reported MRD-Negative rate involving 280 patients. Results indicated a pooled MRD-Negative rate of 63% (95% CI, 43%–81%, I^2^ = 89%) (Figure 4). The Egger test (*p* > 0.05) revealed a low probability of publication bias. Among the six studies analysed, three used next-generation flow (NGF) at 10^−5^ sensitivity, one used next-generation sequencing (NGS) at 10^−4^, one employed both NGF (10^−5^) and NGS (unreported sensitivity), and one did not specify the MRD method. Since there was a lack of sufficient data, subgroup analysis was not conducted.

### 3.4. Safety assessment

Intervention groups of four RCTs and nine single-arm trials reported any grade 3–4 AEs rate among 542 participants. The pooled rate for grade 3–4 AEs reached 36% (95% CI, 30%–43%, I^2^ = 61%) (Figure 5). The Egger test (*p* > 0.05) revealed a low probability of publication bias. Since the extracted data did not contain 0 or 1, we performed a sensitivity analysis by the method ‘leave one out’ and the results showed stability (Figure S9). In sub-group analysis, we observed that the combination therapy was associated with a higher likelihood of any grade 3–4 AEs than monotherapy (42% vs. 35%). The rest of the results are shown in Table 2. In contrast, the control groups of 3 RCTs showed a 25% incidence of any grade 3–4 AEs among 167 participants (95% CI, 0%–50%, I^2^ = 92%).

Twelve studies reported grade 3–4 infection rate among 551 patients. An 8% rate of grade 3–4 infection was observed in the pooled data (95%CI, 6%–11%, I^2^ = 0%).

Nine studies reported grade 3–4 neutropenia rate among 479 patients. An 18% rate of grade 3–4 neutropenia was observed in the pooled data (95%CI, 13%–23%, I^2^ = 53%).

Eight studies reported grade 3–4 skin rash rate among 435 patients. A 5% rate of grade 3–4 skin rash was observed in the pooled data (95%CI, 1%–9%, I^2^ = 67%).

Seven studies reported grade 3–4 lymphocytopenia rate among 289 patients. A 10% rate of grade 3–4 lymphocytopenia was observed in the pooled data (95%CI, 2%–22%, I^2^ = 86%).

Eleven studies reported grade 3–4 fatigue rate among 513 patients. A 5% rate of grade 3–4 fatigue was observed in the pooled data (95%CI, 3%–8%, I^2^ = 29%).

Six studies reported grade 3–4 hyperglycemia rate among 310 patients. A 4% rate of grade 3–4 hyperglycemia was observed in the pooled data (95%CI, 2%–7%, I^2^ = 6%).

### 3.5. High-risk SMM

We performed subgroup analysis in 11 studies involving 522 high risk patients. It was found that early treatment can better enhance the clinical response for HRSMM patients. HRSMM patients demonstrated a higher pooled PFS rate-12m of 97%, OS rate-12m of 99%, ORR of 77%, CRR of 24% and MRD-Negative rate of 66% compared to all SMM patients of other studies, which reported the pooled PFS rate-12m of 91%, OS rate-12m of 99%, ORR of 53%, CRR of 5% and MRD-Negative rate of 76% (Figure S10-14). The assessment of safety data revealed a higher rate of grade 3–4 any AEs in HRSMM patients (39%) than in all SMM patients (35%). It indicated that the toxicity of early treatment in HRSMM patients was manageable and did not appear to outweigh the benefits of treatment.

## 4. Discussion

SMM is a heterogeneous disease and may not progress over a long period of time. Based on current risk stratification models, the prevalence of SMM in people aged 40 years or over 40 is 0.5%, and approximately one-third of the patients are at moderate or high risk of progression to MM [36]. However, the standard of care for the management of SMM is optimal waiting until progression. The rational reasons are that besides the free of progression in some patients mentioned above, early treatments such as melphalan [7,37,38] and bisphosphonate therapy did not have significant antitumor effects [39–41], and many adverse effects occurred during treatment such as myelodysplasia, renal failure, acute leukemia and osteonecrosis of the jaw. The patients involved in these clinical trials included low, intermediate and high risk of progression; however, the efficacy of treatment may vary for patients with different risk stratifications. Some recent researches have shown that early treatment can provide substantial benefits to patients especially to HRSMM patients, potentially altering the current standard of care. In addition to risk stratification, treatment decisions may be modified by patient comorbidities, financial constraints and patterns of treatment resistance [11]. Our research evaluated the efficacy and toxicity of novel agents, aiming to provide evidence for their clinical implementation. The analysis results indicated that early intervention strategies showed promising outcomes. Furthermore, according to a sub-group analysis of HRSMM patients, we found that treatment regimens can better enhance the clinical response for HRSMM patients.

This meta-analysis observed that early treatment may potentially delay disease progression and enhance the clinical response for SMM patients, as evidenced by the improvement in PFS rate-12m and ORR with early treatment for SMM. We identified high heterogeneity in the analytical process. To address this heterogeneity, we employed a random-effects model to account for between-study variability and conducted subgroup analysis to explore the potential effect modifiers. Based on the subgroup analysis findings, combination therapy and therapies with the aim of cure performed better in PFS rate-12m, ORR and CRR data. This advantage underscores the potential of combination regimens to achieve more profound disease control. The synergy between agents with different mechanisms of action (e.g. IMiDs and mAbs) can inhibit proliferation and induce apoptosis through distinct pathways, thereby inducing deeper clinical responses. Focusing on a certain class of drugs, IMiDs demonstrated a superior improvement in PFS rate-12m and ORR compared with mAbs. These findings suggest that such treatment strategies may be more effective in controlling disease and achieving better clinical outcomes, though this interpretation should be considered in the context of study limitations and heterogeneity. In the research conducted by Mateos et al. [18], an assessment of the subsequent treatments showed that early treatment with lenalidomide plus dexamethasone did not induce more resistant relapses. Furthermore, therapies with the aim of cure could significantly increase the MRD negative rate, which relates to longer PFS and OS [42]. Our analysis did not find any significant promotion of OS rate-12m. This lack of improvement in OS rate-12m could be due to several factors, including insufficient sample size, the relatively low early mortality rate of SMM and insufficient follow-up time leading to missing OS data. However, two RCTs [18,19] of lenalidomide-based treatment regimens indicated significant improvements in OS, demonstrating that early intervention could provide survival benefits. A recent study evaluated the effectiveness of various treatment options in patients with SMM, and by comparing their efficacy with that of newly diagnosed MM (NDMM) patients, revealed no compelling evidence that early treatment is more effective in SMM compared to NDMM [5]. This discrepancy might stem from methodological differences, including distinct patient cohorts and outcome assessments. More comprehensive research is necessary to reconcile these findings and clarify the role of early treatment in SMM. In the analysis of assessing safety data, the pooled prevalence for grade 3–4 AEs reached was 36% with 95% CI 30%–43%, there was a trend for increased incidence compared with the data from the RCTs control group. In sub-group analysis, patients receiving combination therapy were more likely to experience any grade 3–4 adverse events compared to monotherapy. In addition, we also extracted data on infection, neutropenia, skin rash, lymphocytopenia, fatigue and hyperglycemia. Pooled data revealed neutropenia (18%), lymphocytopenia (10%) and infection (8%) as the predominant grade 3–4 adverse events. Lonial et al. demonstrated that therapy with lenalidomide did not adversely affect quality of life, which provided the rationale for using lenalidomide treatment for SMM patients from a safety perspective. Unfortunately, only a very limited number of studies assessed quality of life data, which was insufficient for a comprehensive analysis. Moving forward, it will be important to report quality-of-life data in future trials to ensure that intervention on SMM patients is valuable and reliable.

According to the Mayo 2018 [16], patients who evolved into the high-risk category during follow-up have an approximately threefold higher risk of progression than patients who remained in the low- or intermediate-risk category. Furthermore, patterns and numbers of mutations between HRSMM and MM are similar, suggesting that HRSMM may be a distinct group of asymptomatic patients who may benefit from early intervention [43]. In our sub-group analysis of HRSMM, early treatment may improve the clinical response in HRSMM patients. This is specifically reflected in the ORR and CRR data from our statistical results. It indicated that patients with HRSMM may be able to gain greater benefits from the treatment. MRD negativity typically correlates with significantly improved PFS and deeper treatment response [42], but our analysis did not directly prove this due to the lack of MRD-negative data in the control group of RCTs. It was noteworthy that among the studies that included HRSMM patients, more studies used intensive drug regimens compared to studies that included all SMM patients. However, due to the limitations imposed by the number of eligible articles, it was not possible to perform a further sub-group analysis based on the intensity of the treatment. Therefore, this might affect the validity of our conclusions. Notably, regarding clinical management recommendations for HRSMM, the 2026 NCCN guidelines prioritize enrollment in clinical trials as the preferred option, followed by observation or single-agent therapy for selected patients [44]. In addition, there are some other points worth considering in the above conclusions. First of all, the limited concordance among different risk stratification models in identifying high-risk patients significantly complicates the interpretation and comparison of clinical trial data for SMM patients across studies employing distinct models. Furthermore, the diagnostic criteria of multiple myeloma (MM) has changed in 2014, and it classified SMM patients with a heightened risk of progression (defined as BMPC involvement of ≥60%, involved to uninvolved serum FLC ratio of ≥100, and > 1 focal bone lesions on magnetic resonance imaging [MRI]) [2] as ‘active’ MM. Due to stage migration, the results from clinical trials may be susceptible to the Will Rogers phenomenon [45]. The majority of the research included in our assessment employed Mayo 2008 or PETHEMA 2007 risk stratification model; therefore, the selected HRSMM patients involved the above ultrahigh-risk SMM patients and the analysis results may be affected. Combining the above analysis with the current literature review, current risk stratification models are not accurate enough to identify truly high-risk SMM patients. Hence, more and better-designed RCTs and risk models are needed in the future, in order to identify patients who would benefit most from early treatment.

Our meta-analysis, which systematically evaluates treatment efficacy and safety in SMM by encompassing both RCTs and NRSIs, benefits from its comprehensive literature coverage that includes these study types. By incorporating a larger sample size for evaluation, we increased the precision and accuracy of the estimates and enhanced the statistical power. Moreover, we analysed many novel agents during the treatment for SMM such as Lenalidomide, Daratumumab, Isatuximab, Elotuzumab, Carfilzomib and Siltuximab and obtained more comprehensive data, allowing for a more objective evaluation of existing treatments. However, several limitations of this meta-analysis and the applicability of its conclusions should be considered in our study. First of all, the observed high degree of heterogeneity, which arises from methodological distinctions between controlled clinical trials and single-arm studies, divergent patient selection criteria and treatment regimens, may diminish confidence in pooled effect sizes and the generalizability of conclusions. Adding to this concern, these inherent design constraints may explain the potential publication bias detected in efficacy assessments. Second, the limited sample sizes of some studies with low statistical power influenced the reliability of the analysis results. Finally, because of the insufficient duration of follow-up and the scarcity of reported details of adverse events in some trials, the analysis of safety may be influenced.

## 5. Conclusion

In summary, this meta-analysis suggests that SMM patients, especially HRSMM patients, might benefit from early treatment in delaying disease progression. However, in applying these results to clinical work, adverse events and the cost-effectiveness of the regimens should be considered carefully. Further, more RCTs with the endpoint of OS and health-related quality of life are essential to answer whether early treatment has more benefits than harms for SMM patients. Last but not least, unified risk models and enrollment criteria for future clinical trials are also required.

## Supplementary Material

Supplemental Material

## Data Availability

The original data in the study are included in this article and its supplementary material files. Further inquiries are available from the corresponding author on request.
