# Supplementary material for: Efficacy and toxicity of treatment of smoldering multiple myeloma: a systematic review and meta-analysis
Source: Ann Med. 2025 Sep 24;57(1):2560679. doi: 10.1080/07853890.2025.2560679 (PMC12466189; doi:10.1080/07853890.2025.2560679)
Supplement: Supplemental Material [file IANN_A_2560679_SM7796.zip › suppl_data/Supplementary tables.docx]

| **Table S1. Original data of studies included in the meta-analysis.** | | | | | | | |
| --- | --- | --- | --- | --- | --- | --- | --- |
| **Study, year** | **Treatment arm /Control arm** | **Survival outcomes** | | **Clinical response** | | **Grade 3-4 AEs(%)** | **MRD Negativity  Rate(%)** |
|  |  | **OS rate-12m (%)** | **PFS rate-12m (%)** | **ORR(%)** | **CRR(%)** |  |  |
| Witzig et al, 2013 | Thalidomide+Zoledronic acid / Zoledronic acid | 97.1/96.9(1-year) | 86/55(1-year) | 37/0 | 0/0 | 49/39 | NA |
| Mateos et al,2016 | LenDex/Observation | 98.5/95.4(1-year) | 98.1/78.9(1-year) | 79/0 | 14/0 | 26/NA | NA |
| Lonial et al,2020 | Lenalidomide/Observation | 98.9/100(1-year) 97.8/100(2-year) 97.8/95(3-year) | 98/89(1-year) 93/76(2-year) 91/66(3-year) | 50/0 | 0/0 | 41/4.7 | NA |
|  | Lenalidomide | 98(1-year) 95(2-year) 95(3-year) | 95(1-year) 87(2-year) 84(3-year) | 47.7 | 2 | 45 | NA |
| Rajkumar et al,2003 | Thalidomide | 96(1-year) 96(2-year) | 80(1-year) 63(2-year) | 66 | 0 | 23 | NA |
| Barlogie et al, 2008 | Thalidomide+Bisphosphonates | 91(4-year) | 60(4-year) | 25 | 5 | NA | NA |
| Brighton et al, 2019 | Siltuximab/Placebo | 93/90(1-year) | 84.5/74.4(1-year) | NA | NA | 44/29 | NA |
| Jagannath et al,2018 | Elotuzumab 20 mg/kg | NA | 79(1-year) 71(2-year) | 13.3 | 0 | 47 | NA |
|  | Elotuzumab 10 mg/kg | NA | 81(1-year) 68(2-year) | 6.3 | 0 | 38 | NA |
| Manasanch et al, 2019A | Isatuximab | NA | NA | 62.5 | 5 | NA | NA |
| Manasanch et al, 2019B | Pembrolizumab | 100(1-year) | 84.6(1-year) | 7.7 | 7.7 | NA | 7.7 |
| Landgren et al,2020 | Daratumumab(Intense) | NA | 95(1-year) 90(2-year) | 56.1 | 4.9 | 43.9 | NA |
|  | Daratumumab(Intermediate) | NA | 88(1-year) 82(2-year) | 53.7 | 9.8 | 26.8 | NA |
|  | Daratumumab(Short) | NA | 84(1-year) 75(2-year) | 37.5 | 0 | 15 | NA |
| Mailankody et al,2022 | IxaDex | NA | NA | 57 | 0 | NA | NA |
| Korde et al,2015 | KRd-R | 100(1-year) | 100(1-year) | 100 | 100 | NA | 92 |
| Liu et al,2018 | EloLenDex | NA | NA | 84 | 6 | NA | NA |
| Mateos et al, 2021 | KRd-ASCT-KRd-Rd | 98.8(1-year) | 100(1-year) | 95 | 64 | NA | 53 |
| Kazandjian et al, 2021 | KRd-R | 100(8-year) | 100(1-year) 100(2-year) | 100 | 75.9 | 38.9 | 70.4 |
| Sklavenitis-Pistofidis  et al,2022 | EloLenDex | 100(1-year) 95.6(4-year) | 100(1-year) 88.7(4-year) | 87 | 9 | NA | NA |
| Kumar et al,2022 | DKRd-DKRd-DR | NA | 89.9(3-year) | 94 | 63 | NA | 84 |
| Nadeem et al,2023 | DRVd | NA | NA | 87 | 40 | NA | 58 |
| Manasanch et al,2023 | IsaLen | NA | NA | 89 | 0 | 47 | NA |
| Abbreviations: AEs, adverse events; ASCT, autologous stem cell transplantation; CRR, complete response rate; DKRd, daratumumab-carfilzomib-lenalidomide-dexamethasone; DRVd, daratumumab-lenalidomide-bortezomib-dexamethasone; EloLenDex, elotuzumab-lenalidomide-dexamethasone; IsaLen, isatuximab-lenalidomide; IxaDex, ixazomib-dexamethasone; KRd,carfilzomib-lenalidomide-dexamethasone; LenDex, lenalidomide-dexamethasone; MRD, minimal residual disease; NA, not available; ORR, overall response rate; OS, overall survival; PFS, progression-free survival. | | | | | | | |

**Table S2.** **Quality assessment of non-randomized studies.**

| Study | Q1 | Q2 | Q3 | Q4 | Q5 | Q6 | Q7 | Q8 | Total |
| --- | --- | --- | --- | --- | --- | --- | --- | --- | --- |
| Rajkumar et al,2003 | 2 | 2 | 2 | 2 | 1 | 2 | 2 | 0 | 13 |
| Barlogie et al, 2008 | 2 | 2 | 2 | 2 | 1 | 2 | 2 | 0 | 13 |
| Korde et al,2015 | 2 | 2 | 2 | 2 | 0 | 1 | 2 | 0 | 11 |
| Jagannath et al,2018 | 2 | 2 | 2 | 2 | 1 | 2 | 2 | 0 | 13 |
| Liu et al,2018 | 2 | 2 | 2 | 2 | 0 | 0 | 2 | 0 | 10 |
| Manasanch et al, 2019A | 2 | 2 | 2 | 2 | 0 | 0 | 2 | 0 | 10 |
| Manasanch et al, 2019B | 2 | 2 | 2 | 2 | 0 | 1 | 2 | 0 | 11 |
| Landgren et al,2020 | 2 | 2 | 2 | 2 | 1 | 2 | 2 | 2 | 15 |
| Mateos et al, 2021 | 2 | 2 | 2 | 2 | 1 | 2 | 2 | 2 | 15 |
| Kazandjian et al, 2021 | 2 | 2 | 2 | 2 | 2 | 2 | 2 | 2 | 16 |
| Mailankody et al,2022 | 2 | 2 | 2 | 2 | 0 | 2 | 2 | 0 | 12 |
| Kumar et al,2022 | 2 | 2 | 2 | 2 | 0 | 2 | 2 | 0 | 12 |
| Nadeem et al,2023 | 2 | 2 | 2 | 2 | 1 | 2 | 2 | 1 | 14 |
| Manasanch et al,2023 | 2 | 2 | 2 | 2 | 0 | 0 | 2 | 0 | 10 |

**Note:** Q1: A clearly stated aim. Q2: Inclusion of consecutive patients. Q3: Prospective collection of data. Q4: Endpoints appropriate to the aim of the study. Q5: Unbiased assessment of the study endpoint. Q6: Follow-up period appropriate to the aim of the study. Q7: Loss to follow up less than 5%. Q8: Prospective calculation of the study size.

Supplementary materials Figures Caption

Figure S1. Quality assessment of RCTs

Figure S2 - S 1 4 To enhance readability, within the meta -analysis figures, we designated the literature by the treatment arm of each study. Lenalidomide (A) and (B) were used to denote RCT and the single -arm study within the Lonial et al., 2020 research, respectively. EloLenDex (A) and (B) were utilized to represent the studies conducted by Liu et al., 2018, and Sklavenitis -Pistofidis et al., 2022, respectively. KRd -R(A) and (B) were designated to reference the research articles authored by Korde et al. in 2015 and Kazandjian et al. in 2021.

Figure S2. Subgroup analysis by treatment purpose of PFS rate -12m of treatment in SMM.

Figure S3. Subgroup analysis by treatment regimen of PFS rate -12m of treatment in SMM (monotherapy vs. combination therapy.

Figure S4. Subgroup analysis by study type of OS rate -12m of treatment in SMM.

Figure S5. Subgroup analysis by treatment purpose of OS rate -12m of treatment in SMM.

Figure S6. Subgroup analysis by treatment regimen of OS rate -12m of treatment in SMM. (A) Subgroup analysis by treatment regimen (monotherapy vs. combination therapy) of OS rate -12m of treatment in SMM. (B) Subgroup analysis by treatment regimen (IMiDs vs. mAbs) of OS rate -12m of treatment in SMM. Within the monotherapy group, treatment regimens were further subdivided into immunomodulatory drugs and monoclonal antibodies.

Figure S7. Subgroup analysis by treatment purpose of CRR of treatment in SMM.

Figure S8. Subgroup analysis by treatment regimen of CRR of treatment in SMM (monotherapy vs. combination therapy) .

Figure S9 . Sensitivity analysis using the leave -one -out test for any grade 3 -4 AEs rate.

Figure S10. Subgroup analysis by disease of PFS rate -12m rate of treatment in SMM or HRSMM.

Figure S11. Subgroup analysis by disease of OS rate -12m of treatment in SMM or HRSMM.

Figure S12. Subgroup analysis by disease of ORR of treatment in SMM or HRSMM.

Figure S13. Subgroup analysis by disease of CRR of treatment in SMM or HRSMM.

Figure S14. Subgroup analysis by disease of MRD -Negative rate of treatment in SMM or HRSMM.
