# Supplementary figures and images for: Efficacy and toxicity of treatment of smoldering multiple myeloma: a systematic review and meta-analysis
Source: Ann Med. 2025 Sep 24;57(1):2560679. doi: 10.1080/07853890.2025.2560679 (PMC12466189; doi:10.1080/07853890.2025.2560679)

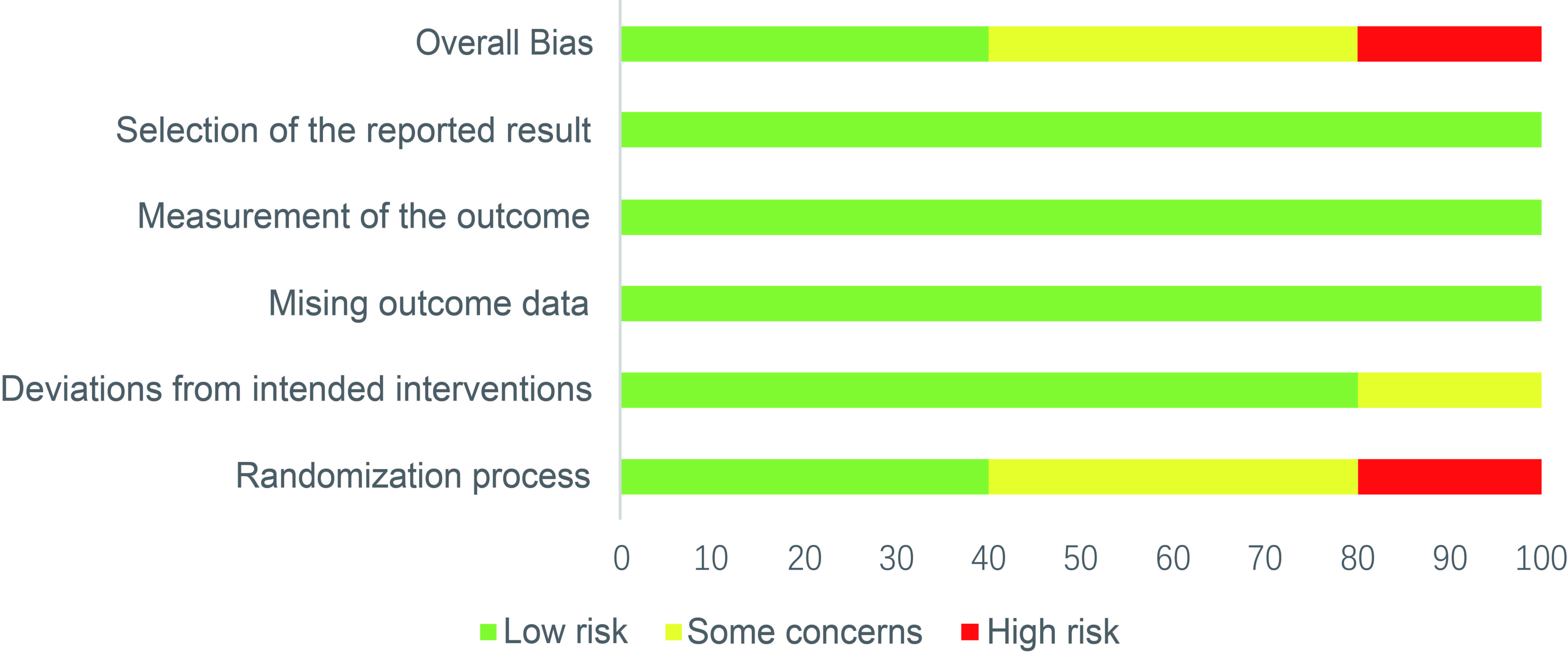

Supplement: Supplemental Material [file IANN_A_2560679_SM7796.zip › suppl_data/Figure S1.jpg]

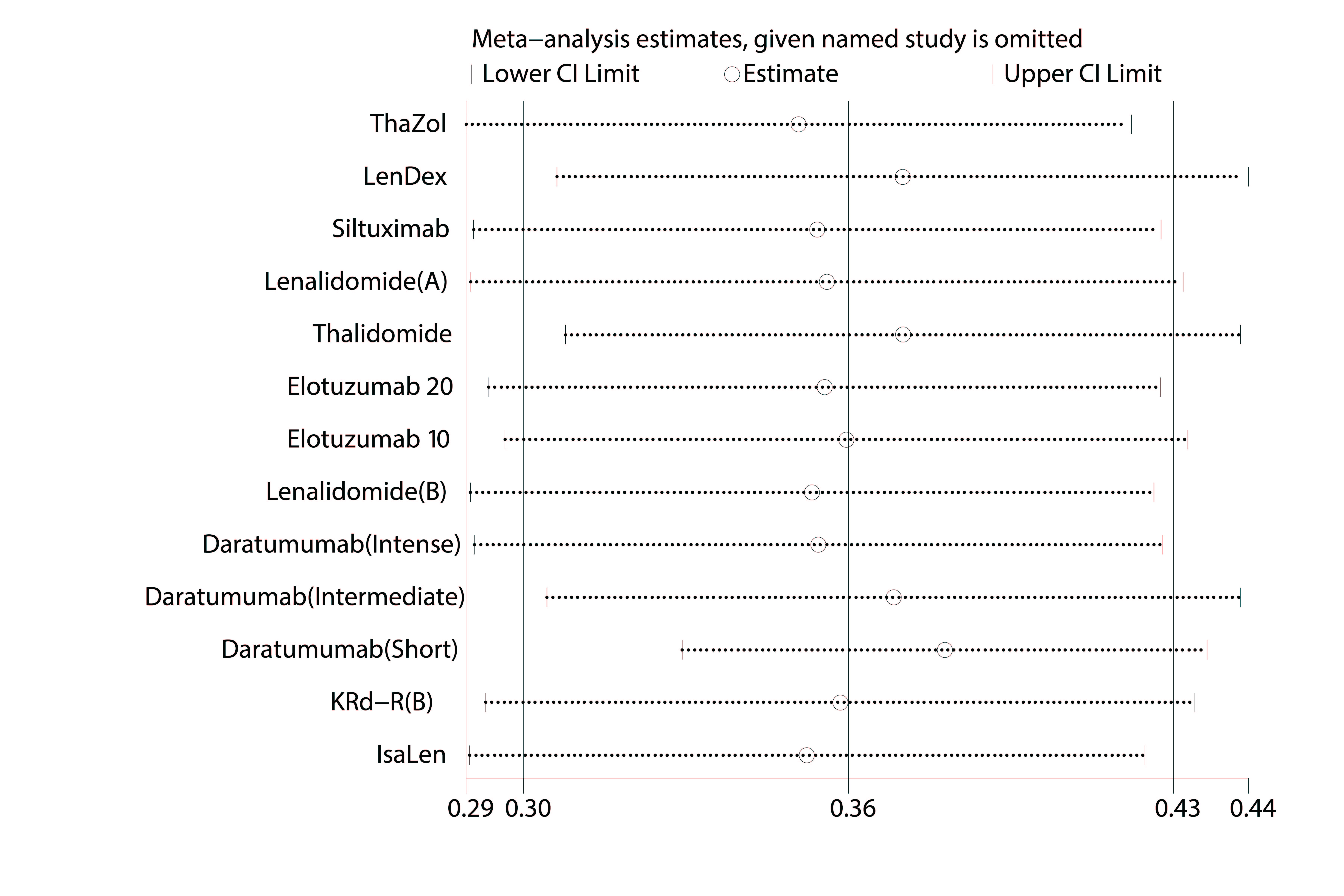

Supplement: Supplemental Material [file IANN_A_2560679_SM7796.zip › suppl_data/Figure S9.jpg]
